# Chromosomal mapping of repetitive DNA in *Melipona seminigra merrillae* Cockerell, 1919 (Hymenoptera, Apidae, Meliponini)

**DOI:** 10.3897/CompCytogen.v15i1.56430

**Published:** 2021-03-19

**Authors:** Ingrid Cândido de Oliveira Barbosa, Carlos Henrique Schneider, Leonardo Gusso Goll, Eliana Feldberg, Gislene Almeida Carvalho-Zilse

**Affiliations:** 1 Grupo de Pesquisas em Abelhas, Programa de Pós-Graduação em Genética, Conservação e Biologia Evolutiva, Instituto Nacional de Pesquisas da Amazônia, Av. André Araújo 2936, Petrópolis, 69067-375, Manaus, Brazil; 2 Laboratório de Pesquisa em Ciências Médicas, Universidade Federal da Integração Latino Americana, Av. Silvio Américo Sasdelli 1842, Itaipu A, 85866-000, Foz do Iguaçu, Brazil; 3 Instituto de Natureza e Cultura – INC, R. Primeiro de Maio s/n, Colônia, 69630-000, Benjamin Constant, Brazil; 4 Laboratório de Genética Animal, Programa de Pós-Graduação em Genética, Conservação e Biologia Evolutiva, Instituto Nacional de Pesquisas da Amazônia, Av. André Araújo 2936, Petrópolis, 69067-375, Manaus, Brazil; 5 Grupo de Pesquisas em Abelhas, Coordenação de Biodiversidade, Instituto Nacional de Pesquisas da Amazônia, Av. André Araújo 2936, Petrópolis, 69067-375, Manaus, Brazil

**Keywords:** Cytogenetics, fluorescence in situ hybridization (FISH), heterochromatin, stingless bee

## Abstract

*Melipona* Illiger, 1806 is represented by 74 known species of stingless bees, distributed throughout the Neotropical region. Cytogenetically it is the most studied stingless bee genus of the tribe Meliponini. Member species are divided in two groups based on the volume of heterochromatin. This study aim was to analyze the composition and organization of chromatin of the stingless bee subspecies *Melipona seminigra merrillae* Cockerell, 1919 using classical and molecular cytogenetic techniques, so contributing to a better understanding of the processes of chromosomal changes within the genus. We confirm that *M. seminigra merrillae* has a chromosome number of 2n = 22 and n = 11, results that differ from those reported for the genus in the absence of B chromosomes. The heterochromatic pattern revealed a karyotype composed of chromosomes with a high heterochromatin content, which makes it difficult to visualize the centromere. Silver nitrate impregnation (Ag-NOR) showed transcriptionally active sites on the second chromosomal pair. Staining of base-specific fluorophores DAPI-CMA_3_ indicated a homogeneous distribution of intensely DAPI-stained heterochromatin, while CMA_3_ markings appeared on those terminal portions of the chromosomes corresponding to euchromatin. Similar to Ag-NOR, fluorescence in situ hybridization (FISH) with 18S ribosomal DNA probe revealed distinct signals on the second pair of chromosomes. Microsatellite mapping (GA)_15_ showed markings distributed in euchromatic regions, while mapping with (CA)_15_ showed marking patterns in heterochromatic regions, together with a fully marked chromosome pair. Microsatellite hybridization, both in heterochromatic and euchromatic regions, may be related to the activity of transposable elements. These are capable of forming new microsatellites that can be dispersed and amplified in different regions of the genome, demonstrating that repetitive sequences can evolve rapidly, thus resulting in within-genus diversification.

## Introduction

Bees of the genus *Melipona* Illiger, 1806 are highly social insects, with collective offspring care, division into castes and one or more overlapping generations between adult colony members also within castes there are fully reproductive, poorly reproductive and sterile individuals (Wilson and Hölldobler 2005; Michener 2007). Members of the tribe Meliponini are commonly called stingless bees, and their species are distributed throughout the Neotropical region. The Meliponini tribe comprise 33 genera with approximately 417 known species (Camargo and Pedro 2013). Of these genera, *Melipona* is the most species-rich represented by 76 valid species, of which 43 occur in Brazil (Camargo and Pedro 2013; Pedro 2014).

With 23 species with described karyotypes, *Melipona* has the largest number of cytogenetically studied members (Tavares et al. 2017). According to karyotypic analyses, most species of the genus have a chromosome number of 2n = 18 (queens/workers) and n = 9 (drones). However, there are some exceptions. The species *Melipona quinquefasciata* Lepeletier, 1836 and *M. rufiventris* Lepeletier, 1836 which have B chromosomes (Rocha et al. 2007; Lopes et al. 2008), while *M. seminigra merrillae* Cockerell, 1919, *M. seminigra pernigra* Moure & Kerr, 1950 and *M. seminigra abunensis* Cockerell, 1912 have 2n = 22 and n = 11 chromosomes (Francini et al. 2011; Andrade-Souza et al. 2018; Cunha et al. 2018). *Melipona* has a unique distribution pattern of constitutive heterochromatin (CH), which differentiates it from other Meliponini (Hoshiba and Imai 1993; Rocha et al. 2003; Cunha et al. 2018). Based on the distribution pattern/quantity of CH, species in the genus can be divided into two groups: Group I – composed of species with a low amount of CH, present only in pericentromeric regions, and Group II – composed of species with a high amount of CH, present along almost the entire length of each chromosome (Rocha and Pompolo 1998; Rocha et al. 2002; Lopes et al. 2011).

The objective of this study was to use a combination of classical cytogenetics and molecular tools to obtain information on the composition and organization of the chromatin of *Melipona seminigra merrillae*, an Amazonian stingless bee.

## Material and methods

Larvae of *M. seminigra merrillae* were collected in colonies maintained in the Instituto Nacional de Pesquisas da Amazônia (INPA) Meliponary, Manaus, Amazonas, Brazil. Mitotic chromosomes were obtained using the protocol given by Imai et al. (1988) with modifications: cerebral ganglia were removed from larvae in the post-defecation stage and dissected in 1% sodium citrate solution containing 0.005% colchicine. The cerebral ganglia were then dissected using entomological pins to expose cellular contents. The material containing metaphasic chromosomes were mounted on air-dried slides, which had been previously treated with three sequential fixatives: first (water: ethanol: acetic acid, 4:3:3), second (ethanol: acetic acid, 1:1), third (100% acetic acid). Slides were then stained with 5% Giemsa solution in Sörensen buffer (0.06 M, pH 6.8) for 20 minutes.

To analyze constitutive heterochromatin, slides with chromosome-bearing material were subjected to the C-banding technique, using Sumner’s (1972) protocol with increased treatment time. Slides were treated in 0.2 M hydrochloric acid (HCl) solution for 6 minutes, washed in distilled water and incubated for 9 minutes in 5% barium hydroxide solution freshly prepared, filtered and maintained at 60 °C. Barium hydroxide action was halted by immersing slides for 1 minute in 0.2 M HCl solution at room temperature. Slides were then incubated in 2xSSC solution (sodium chloride 0.3M and 0.03M trisodium citrate, pH 7.0) in a water bath at 60 °C for 12 minutes, washed in running water and then stained with 5% Giemsa solution in Sörensen buffer (0.06 M, pH 6.8).

The active Nucleolus Organizer Regions (NORs) were detected with silver nitrate impregnation (Ag-NOR), following the protocol proposed by Howell and Black (1980). Sequential staining with fluorochromes chromomycin A_3_ (CMA_3_) and 4’,6-diamidino-2-phenylindole (DAPI) was carried out following the methodology of Schweizer (1980).

Fluorescence *in situ* hybridization (FISH) was performed following Pinkel’s et al. (1986) protocol. Products obtained via PCR (18S ribosomal DNA probe) were labeled by biotin-14-dATP nick translation (Biotin Nick Translation mix; Invitrogen) and digoxigenin11-dUTP nick (Dig-Nick Translation mix; Roche Applied Science) following the manufacturer’s instructions. This probe was obtained by PCR amplification using the primers 18SF1 (5’-GTCATATGTTGTCTCAAAGA-3’) and 18SF2 (5’ – TCT AAT TTT TTC AAA GAT AAC GC – 3’) designed for *Melipona quinquefasciata* (Pereira 2006). The PCR reaction was performed in a thermocycler with a final volume of 20 μL (2 μL of dye 10X + 1.2 μL of MgCl_2_ 25 mM + 0.2 μL of dNTPs + 1 μL of primer 18SF1 + 1 μL of primer 18SF2 + 1 μL of DNA template + 13.4 μL of milli-Q water). The amplification cycle had the following steps: 3 minutes at 94 °C (initial denaturation), 1 minute at 95 °C (denaturation), 1 minute at 55 °C (priming cycle), 2 minutes at 72 °C (extension), 5 minutes at 72 °C (final extension). The microsatellites (GA)_15_, (CA)_15_ were labelled directly with Cy3 in the 5′ regions (Sigma, St. Louis, MO, USA).

Images of metaphase chromosomes were captured with a Leica DM 2000 epifluorescence photomicroscope, using a 100× immersion objective. Slides stained with fluorochromes (CMA_3_ and DAPI) were analyzed using 450–480 nm (CMA_3_) and 330–385 nm (DAPI) excitation filters. Adobe Photoshop 7.0 CS4 software was used to assemble karyotype images of mitotic metaphase chromosomes. Each chromosome was virtually cut and paired according to its size, following a decreasing order of size. In this study, 240 larvae were analyzed using 10 metaphases for each individual, and about 40 individuals produced satisfactory results.

## Results

**Figure 1.**
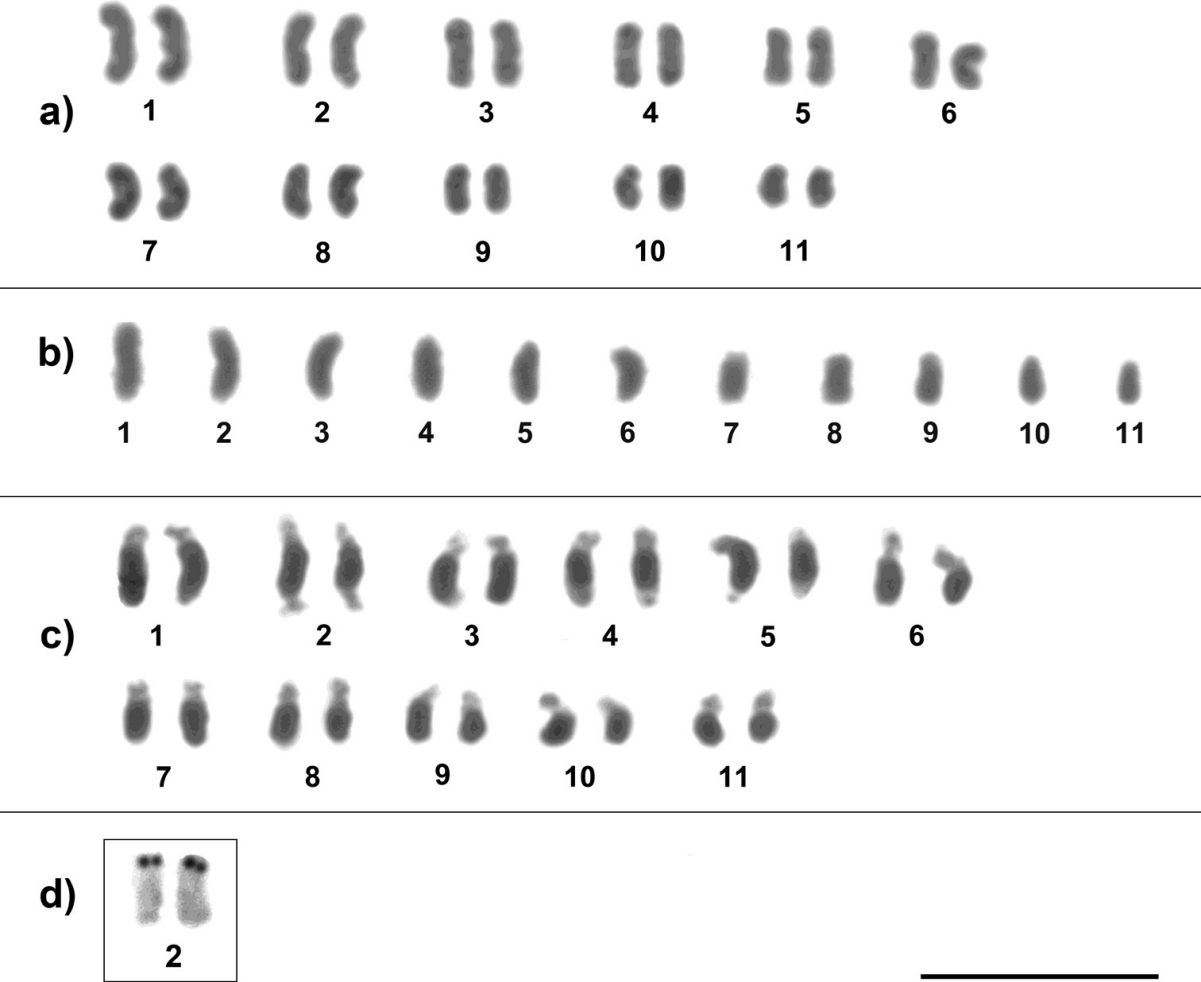
Representative karyotype of *Melipona seminigra merrillae* with Giemsa-stained chromosomes **a** female karyotype with 2n = 22 **b** male karyotype with n = 11 **c** C-banding **d**Ag-NOR-banding of the second chromosome pair. Scale bar: 10 μm.

After analysis, we found that *M. seminigra merrillae* presented chromosomal numbers 2n = 22 and n = 11 (Fig. 1a, b). C-banding technique revealed a karyotype with a high heterochromatic content for all chromosomes, making it difficult to accurately visualize the position of the centromere. Therefore, the morphological identification was less precise or even impossible (Fig. 1c). Silver nitrate impregnation (Ag-NOR) in *M. seminigra merrillae* showed transcriptionally active ribosomal sites on the second pair of chromosomes (Fig. 1d).

**Figure 2.**
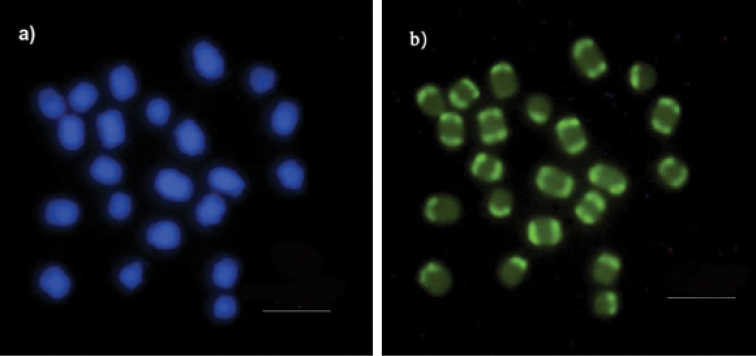
Fluorochrome staining of *Melipona seminigra merrillae* chromosomes **a**DAPI, with uniform staining on almost every chromosome **b** CMA_3_, showing euchromatin in terminal regions of all chromosomes. Scale bars: 10 μm.

**Figure 3.**
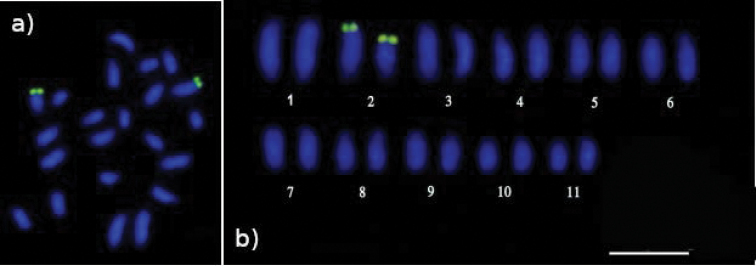
Distribution pattern of 18S rDNA sites on *Melipona seminigra merrillae* chromosomes. Additionally, size heteromorphism between homologues is also evident in the second pair **a** metaphase plate **b** karyotype with paired chromosomes. Scale bar: 10 μm.

Regarding base-specific fluorophores, DAPI stained almost the entire length of all chromosomes evenly, except for the weakly stained terminal regions (Fig. 2a). In contrast, CMA_3_ marked terminal regions (Fig. 2b) 18S ribosomal DNA sequence mapping showed two terminal markers on the second chromosomal pair, with a difference in size between homologues (Fig. 3).

**Figure 4.**
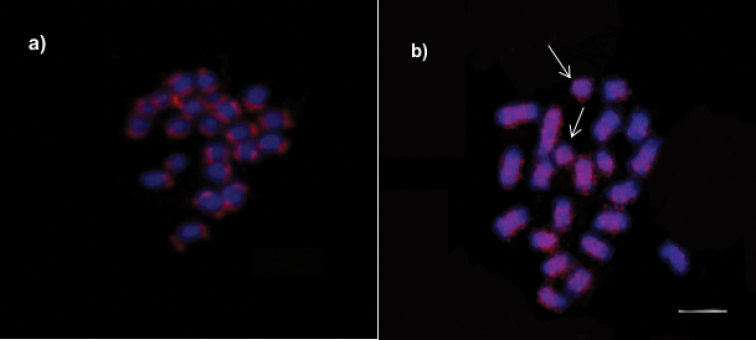
Repetitive DNA mapping on *Melipona seminigra merrillae* chromosomes **a** probe (GA)_15_ hybridized with euchromatic regions and **b** (CA)_15_ hybridized with heterochromatic regions. Arrows indicate fully marked chromosomes. Scale bar: 10 μm.

The microsatellite probe (GA)_15_ labeled only euchromatic regions (Fig. 4a), while (CA)_15_ revealed signals spread almost along the entire length of all chromosomes except for terminal regions; however, a particular chromosome pair was fully labeled (Fig. 4b).

## Discussion

Our findings confirm the observations of Francini et al. (2011) on the chromosome number of *M. seminigra merrillae*. Similar karyotype numbers have been reported for *M. seminigra abunensis* and *M. seminigra pernigra* (Andrade-Souza et al. 2018; Cunha et al. 2018). Such results differ from those already described for the genus, which generally has 2n = 18 and n = 9. Chromosome number variations have been also recorded for *M. rufiventris* and *M. quinquefasciata*; however, changes observed in these species were due to the presence of B chromosomes (Rocha et al. 2002; Capoco 2016; Tavares et al. 2017), which were not recorded in *M. seminigra merrillae*. Chromosome number increase in the studied species is due to centric fission with a subsequent addition of heterochromatin (Cunha et al. 2020).

Francini et al. (2011) classified *M. seminigra merrillae* as belonging to the low heterochromatic content group (group I) during previous cytogenetic studies. However, our analyses showed that this species has heterochromatin distributed almost throughout the entire length of chromosomes, with euchromatic regions restricted to the terminal regions. Consequently, the positions of the centromeres are difficult to determine, which supports the categorization of this species in the high heterochromatic content group (group II) (Rocha and Pompolo 1998; Rocha et al. 2002, 2003; Lopes et al. 2006, 2011; Cunha et al. 2018).

According to Tavares et al. (2010), the amount of heterochromatin in the different groups of *Melipona* is directly related to the size of the genome. Thus, group II species tend to have more genomic DNA than species in group I. The chromosome number of *M. seminigra merrillae* is higher (2n = 22). At present, we do not know whether this species has more DNA compared to those with 2n = 18 and the same heterochromatin distribution pattern. These aspects should be a subject of future investigations.

The use of the Ag-NOR staining method to detect active NORs in *Melipona* normally does not show active sites, however, in *M. seminigra merrillae* it was possible to observe these regions which were clearly seen every time in the second chromosome pair. Similar results were also obtained for two other species, *M. asilvai* Moure, 1971 and *M. marginata* Lepeletier, 1836 (Maffei et al. 2001).

Results of chromosome staining by base-specific fluorophores in *M. seminigra merrillae* were similar to that described in a number of other Meliponini species belonging to group II (Rocha et al. 2003; Miranda et al. 2013; Andrade-Souza et al. 2018; Cunha et al. 2018). Several authors have suggested that CMA_3_^+^ markings in Meliponini have a strong correlation with NORs (Rocha et al. 2007; Lopes et al. 2011; Lopes et al. 2012). Studies of the genus *Melipona* frequently report a chromosome pair strongly marked by CMA_3_. However, this was not the case in *M. seminigra merrillae*. Although the nucleolus organizing regions are characterized by rich concentration of CG bases, use of CMA_3_ demonstrates that, in this species, such regions do not always coincide, a fact also reported for other Meliponini (Duarte et al. 2009; Godoy et al. 2013).

Heteromorphism in size of a particular chromosome pair was found among all metaphases analyzed for homologous chromosomes by mapping 18S rDNA sites. Apparently this is a recurrent characteristic in Meliponini (Rocha et al. 2007; Menezes et al. 2014; Andrade-Souza et al. 2018), and it is due to the repetitive nature of ribosomal DNAs, that results in errors during duplication of the genetic material or uneven crossing-over followed by deletion of a small part of the chromosome (Araújo et al. 2002; Mampumbu 2002).

In general, heterochromatic regions of chromosomes are characterized by large amounts of repetitive DNA (Cioffi et al. 2011). Although *M. seminigra merrillae* chromosomes carry large amounts of heterochromatin, the repetitive DNA probe (GA)_15_ hybridized with euchromatic regions, a pattern also observed in chromosome mapping of other Meliponini: in *M. scutellaris* Latreille, 1811 by Piccoli et al. (2018), and in *M. interrupta* Latreille, 1811 by Travenzoli et al. (2019a). This result may be associated with the presence of a family of satellite DNA or even with transposable elements (TEs) that could be linked to gene regulation. Among the bees, TEs have been reported for the genus *Apis* Linnaeus, 1758 (Lampe et al. 2003) and for some *Melipona* species (Cunha et al. 2020).

The presence of positive microsatellite (CA)_15_ signals scattered along the chromosomes of *M. seminigra merrillae* is similar to the pattern revealed by C-banding. Considering that microsatellites, or simple sequence repeats (SSRs), are notable components of constitutive heterochromatin, such repeats probably play an important role in chromosomal organization, regulation of gene expression, dissemination of heterochromatin and, in some cases, in increasing the size of the genome. These functions of SSRs have been demonstrated both for bees and other organisms (Cioffi et al. 2011; Milani and Cabral-de-Melo 2014; Biscotti et al. 2018; Cunha et al. 2020).

Our results indicate that the chromatin of *M. seminigra merrillae* has specific distribution patterns for each type of repeat, a characteristic that may be associated with the occurrence of chromosomal rearrangements in this species. Distribution of SSRs in heterochromatic and euchromatic regions in *Melipona* can also be explained by their relationship with transposable elements, which may have certain sites predisposed to the formation of new microsatellites. This would, in turn, favor dispersion and amplification of these microsatellites between different genomic regions (Milani and Cabral-de-Mello 2014; Travenzoli et al. 2019b).

## Conclusions

Considering the chromosome number and heterochromatic content, our results are similar to those already revealed for other subspecies of *Meliponaseminigra. A*s a result, the nature of the euchromatin, together with distribution of NOR sites and the 18S rDNA, is similar to that in other species of *Melipona* that belong to the group II. This study also highlights the existence of possible chromosomal rearrangements in *M. seminigra merrillae. Finally*, use of the above-mentioned microsatellite probes for mapping repetitive DNA can expand our knowledge of this type of SSRs in Amazonian stingless bees in the future.
